# Mushroom-Derived Bioactive Molecules as Immunotherapeutic Agents: A Review

**DOI:** 10.3390/molecules26051359

**Published:** 2021-03-04

**Authors:** Dinesh K. Patel, Sayan Deb Dutta, Keya Ganguly, Seong-Jun Cho, Ki-Taek Lim

**Affiliations:** 1 Department of Biosystems Engineering, College of Agriculture and Life Science, Kangwon National University, Chuncheon 24341, Korea; dbhu10@gmail.com (D.K.P.); sayan91dutta@gmail.com (S.D.D.); gkeya14@gmail.com (K.G.); 2 Department of Food Science and Biotechnology, College of Agriculture and Life Sciences, Kangwon National University, Chuncheon 24341, Korea; sj.cho@kangwon.ac.kr

**Keywords:** mushroom, antimicrobial, immunomodulatory, β-glucan, and functional food

## Abstract

Mushrooms with enhanced medicinal properties focus on finding such compounds that could modulate the human body’s immune systems. Mushrooms have antimicrobial, antidiabetic, antiviral, hepatoprotective, antitumor, and immunomodulatory properties due to the presence of various bioactive components. β-glucans are the major constituent of the mushroom cell wall and play a significant role in their biological activity. This review described the techniques used in the extraction of the active ingredients from the mushroom. We highlighted the structure of the bioactive polysaccharides present in the mushrooms. Therapeutic applications of different mushrooms were also described. It is interesting to note that mushrooms have the potential sources of many bioactive products that can regulate immunity. Thus, the development of functional medicinal food based on the mushroom is vital for human welfare.

## 1. Introduction

Mushroom, a fungi kingdom member, has drawn significant interest in medicinal applications due to their anti-inflammatory, antimicrobial, antidiabetic, cardiovascular-protective, hepatoprotective, and anticancer potentials. It regulated the immune system, and macrophages, T cells, dendritic cells (DC), natural killer (NK) cells, and hematopoietic stem cell activities by phagocytic activity, generating reactive oxygen species, inflammatory mediators, and cytokines production [1,2,3,4]. Mushroom is a rich source of proteins, fibers, and vitamins. It is challenging to distinguish between edible and medicinal mushrooms because many edible mushrooms have therapeutic properties, and many medicinal mushrooms are also edible [5,6,7,8]. Approximately 1.5–5.0 million fungi species are known globally, and among them, a few hundred species have medicinal properties [9]. *Agaricus bisporus*, *Lentinulla edodes* (shiitake), *Pleurotus ostreatus* (oyster mushroom), *Flammulina velutipes* (golden needle mushroom or enokitake), *Grifola frondosa* (maitake), and *Volvariella volvacea* (straw mushroom) are commonly used mushrooms worldwide [10]. Chitin and β-glucans are two major constituents of the mushrooms’ cell wall, and out of these, the β-glucan, β-(1 → 3), β-(1 → 4), and β-(1 → 6) make mushrooms a vital material to use as therapeutic agents [11,12,13]. It also contains polysaccharides, polysaccharide–protein complexes, polyphenols, terpenoids, agaritine, ergosterol, and selenium in its structure [14]. Table 1 represents the specific constituents present in different mushrooms. Glucan, heterogeneous polysaccharides, is linked with the large numbers of glucose units. β-glucans derived from different sources have different branching, a branch linkage, and a backbone linkage in their structure that affect biological activity. Therefore, it is necessary to identify and characterize β-glucans with better therapeutic and immune-stimulatory activities [15]. Glucan stimulated the defense responses against infections and cancer [16,17,18]. Additionally, glucan reduced the stress and cholesterol level, hypoglycemic effects, and ulcerative colitis improvements [19,20,21]. Phagocytic cells play significant roles in antitumor activity through their phagocytosis, antibody-dependent cell-mediated cytotoxicity (ADCC), and cytokine secretion process. Cytokines and chemokines regulated individuals’ homeostasis via cell differentiation, proliferation, apoptosis, inflammatory reactions, and immune responses [22]. Cytokines are low-molecular-weight proteins and regulate the intensity and duration of the immune response. However, chemokines are small polypeptides and regulate cell migration, adhesion, and activation of the leukocyte. Functional pleiotropy and redundancy are the characteristic features of the generated cytokines that contain interferons, different stimulating and growth factors, and interleukins (IL) [23]. Cytokines may act as a proinflammatory or anti-inflammatory factor, or both, depending on the local conditions of the target cells [24]. IL-1β, TNFα, IL-6, IL-15, IL-17, and IL-18 cytokines exhibited an inflammatory response, whereas IL-4, IL-10, and IL-13 showed anti-inflammatory potential [25]. 

The productions of tumor necrosis factor-α (TNF-α), interleukin-6 (IL-6, IL-1), and interferon-γ (IFN-γ) cytokines have also been observed in the presence of β-glucans by activating the macrophages cells [26,27]. The antitumor activity of mushroom-derived nonstarch polysaccharides (NSPs) is mediated by the immune system of the host rather than direct action on cells [28]. Herein, we briefly discussed the therapeutic potential of commonly used mushroom extract or its derivatives. Mushrooms are enriched with many essential amino acids, polysaccharides, and vitamins necessary for human health. Therefore, mushrooms can be directly used in the human diet to enhance health due to different bioactive compounds.

## 2. Extraction of the Bioactive Components from Mushrooms

It is well-known that mushroom contains several active components, such as essential amino acids, proteins, vitamins and minerals, fiber, and polysaccharides in their fruiting bodies [29]. β-d-glucan polysaccharides and their derivatives extracted from mushrooms showed immunomodulatory responses [4,30]. Mushroom extracts also exhibited antitumor, antimicrobial, and antiviral potentials [31]. Therefore, it is essential to extract these active ingredients from the mushroom bodies to examine their therapeutic potential. The hot aqueous medium and aromatic solvent process are commonly used to extract bioactive polysaccharides from the mushroom. Here, we describe the extraction process of bioactive components by considering some conventional and modern methods.

### 2.1. Hot Water Extraction

In this method, the mushroom bodies are treated with distilled water and ethanol to remove the samples’ contamination. After that, the extraction of polysaccharides is performed by reacting the cleaned and pulverized mushroom with an alkaline solution (4% NaOH). The reaction media are kept at 4 °C for 12 h. The extracted media are separated by centrifugation or filtration, followed by precipitation with ethanol. The precipitated material is isolated and dialyzed via a cellulose bag to remove the excess alkali and other small molecules. The obtained material is separated and freeze-dried for further analysis. In some cases, a hot ethanol solution has been used to extract polysaccharides rather than an alkaline solvent or hot water. This method has been used in *Termitomyces eurhizus*, *Grifola frondosa*, *Pleurotus tuber-regium*, and *Agaricus bisporus* to extract bioactive components [32,33]. 

### 2.2. Organic Solvent Extraction

In this process, a low-boiling-point organic solvent, such as hexane, is selected to extract bioactive polysaccharides. In brief, the tested mushroom is cleaned and dried. The dried mushroom powder is treated with an appropriate organic solvent at room temperature and left for a week. After that, the filtrate is separated from the mother liquor and concentrated by a rotary evaporator. The obtained material is kept at 4 °C in a dark bottle for further applications. This method has been applied in *Grifola frondosa*, *Hericium erinaceum*, *Agaricus blazei*, *Ganoderma lucidum*, *Coriolus versicolor,* and *Phellinus linteus* for the extraction of bioactive components [34].

### 2.3. Soxhlet Method

In this method, the Soxhlet apparatus is attached to the extraction bottle to isolate bioactive components from mushrooms. The aqueous or organic solvents are utilized for the extraction. Here, the solvent is heated up to its boiling point, and the condensed vapors are collected in a flask. This process is repeated several times as desired. This method has been applied in *Bauhinia variegate*, *Sanguisorba officinalis*, and *Tribulus terrestris* to isolate bioactive components [35].

### 2.4. Ultrasound-Mediated Extraction

In this method, the extraction medium is placed in an ultrasound environment to generate the bubble within the solvent. These bubbles interrupt the plant cell wall and facilitate the solvent inside the plant material. The bioactive components come out due to the interruption of the cell wall and penetration of the solvent. This is a very effective extraction method and has several advantages. Here, a shorter extraction time, low amounts of solvent, and energy are required for the extraction [36].

### 2.5. Supercritical Fluid Extraction

Supercritical fluid extraction is an effective and eco-friendly extraction method applied for isolating a wide range of bioactive components. Carbon dioxide is the most suitable solvent isolating lipids, fat, and other nonpolar components. The polar solvents such as ethanol, methanol, and dichloromethane are added to a supercritical fluid to enhance the solubility [37]. A supercritical state is achieved when the temperature and pressure of the fluid surpassed its critical point.

### 2.6. Accelerated Solvent Extraction

The accelerated solvent extraction method is applied to extract various bioactive components under high temperature and pressure without reaching the solvent’s critical point. This method is also known as pressurized fluid extraction (PFE), high-pressure solvent extraction (HPSE), or enhanced solvent extraction (ESE). Methanol, ethanol, or mixtures of the solvents are used in this method. Significantly less time is required to extract bioactive compounds via this method than the others [35].

## 3. Linkage in Glucan

**Figure 1:**
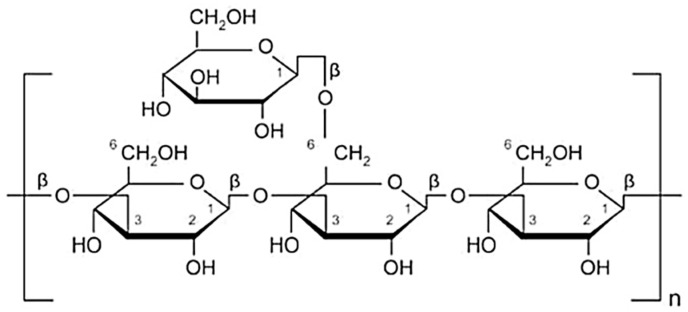
Chemical structure of β-1, 3/1, 6-d-glucan. Glucans are long-chain polysaccharides of a glucose (hexose) monomer, which is linked in the β-1, 3 positions with a smaller side chain of β-1, 6 divaricating from the main polysaccharide chain [29].

Polysaccharides are biologically active biopolymers composed of sugar units such as glucose, galactose, mannose, and fructose in different fashions. The sustainably and economically high yield of polysaccharides is produced from the microbial biomass under controlled fermentation conditions. Various polysaccharides have been extracted from the different mushrooms. The mushroom-derived polysaccharides have received substantial attention due to their superior health benefits activities such as anti-inflammatory, anti-oxidative, immunomodulatory, and hypolipidemic properties [38,39]. Mushroom-extracted bioactive polysaccharides have unparalleled applications in many fields, including food supplements, biomedicine, and cosmetic industries [40]. β-(1 → 3)/β-(1 → 6)-d-glucan and α-(1 → 3)-d-glucan are the most bioactive components present in the mushroom extract, and the structure of β-(1 → 3)/β-(1 → 6)-d-glucan is given in Figure 1. β-glucans have more immunomodulatory potential than α-glucan. Moreover, the α-glucan immunomodulatory activity can be improved by chemical functionalization [41,42].

## 4. Therapeutic Applications of Mushroom

### 4.1. Antitumor Activity

**Figure 2:**
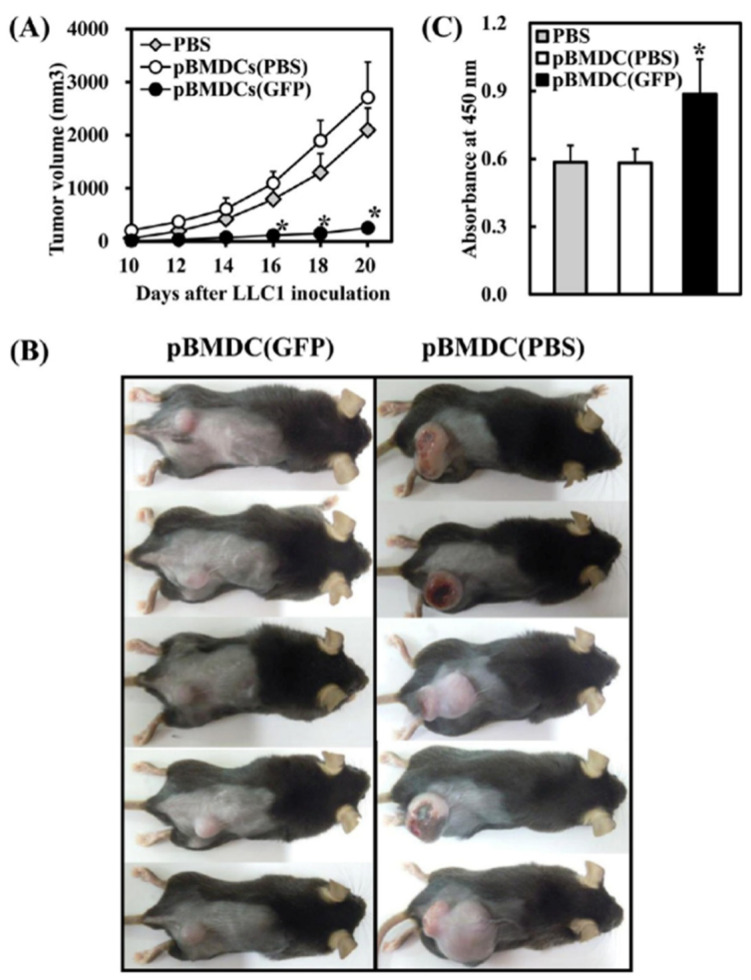
GFP-activated bone-marrow-derived dendritic cells (BMDCs) inhibit tumor growth and increase the level of tumor-antigen-specific antibody production in mice. (**A**) The severity of tumor growth 20 days after LLC1 inoculation. (**B**) Progression of tumor volume from 10 to 20 days after LLC1 inoculation. (**C**) Mice serum was collected to quantify tumor-antigen-specific antibodies by a standard ELISA. Asterisks indicate data that were significantly (* *p* < 0.05) different from those of mice that received PBS or PBS-treated BMDCs [54].

Yeast, grain, mushrooms, ginseng, jimson weed, cinnamon bark, ephedra, garlic, onion, and chamomile are often applied to treat or prevent diseases during the evolution of human history [43,44,45,46,47,48]. Among them, mushrooms have received a considerable amount of interest to use in the biomedical area due to their inheritance properties. Mushrooms are commonly used as a dietary supplement. *Grifola frondosa* (maitake) is an edible mushroom and used as a dietary supplement to promote human health. The maitake extract showed better T-cell activity by accelerating the Th-1 response in tumor-bearing mice and induced the natural killer cell (NK). It also stimulated the dendritic cell (DC) maturation [43,49,50]. Cancer, one of the most severe diseases worldwide, has challenged public health for many years. The extensive use of anticancer drugs showed severe side effects in cancer treatment [51,52,53]. Therefore, the development of an alternative and effective approach is required in the treatment of cancer. Tsao and coworkers extracted the protein (GFP) from maitake and evaluated their antitumor activity [54]. GFP is a nonglucan protein and induced the IFN-γ secretion by murine splenocytes and natural killer cells. It activated the maturation of bone-marrow-derived dendritic cells (BMDCs) via the TLR4-dependent process. A significant improvement in antitumor activity was observed in GFP-treated BMDCs when transferred into tumor-bearing mice via the activation of Th-1 response, showing the immune-modulating potential of GFP. The tumoricidal activity of maitake-extracted GFP is shown in Figure 2. BMDCs from C57BL/6J mice were cultured with tumor-antigen LLC1 lysates and treated with GFP (0.625 μg/mL) or PBS for 24 h. The LLC1-primed cells were then transferred into six-week-old C57BL/6J mice by subcutaneous injection, and a control group was injected with PBS. One week after cell transfer, LLC1 cells were inoculated into the mice at the transfer site. Ten days after LLC1 inoculation, the tumor volume was measured every other day until the end of the experiment on Day 20. All in vitro experiments were done in triplicate (*n* = 3), and the representative results from at least two independent works are given as the mean ± standard deviation (SD). The in vivo work was carried out in pentaplicate (*n* = 5).

**Figure 3:**
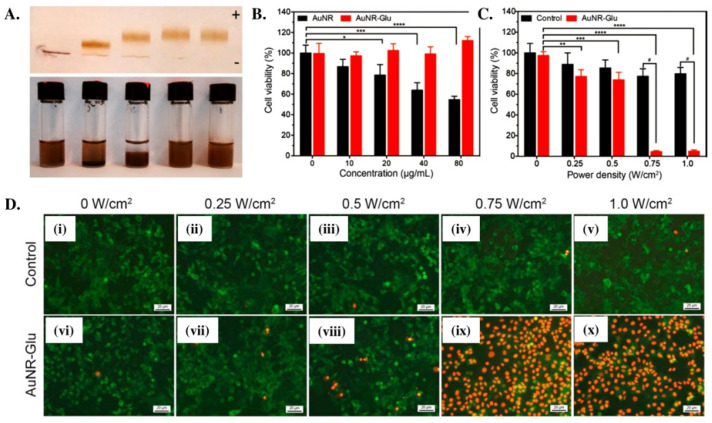
(**A**) β-glucan-modified AuNRs with different concentrations of glucans (0, 1, 5, 10, and 15 mg/mL). (**B**) Cytotoxicity of AuNRs and AuNR-Glu against MCF-7 cells. (**C**) MCF-7 cell viability after photothermal treatment with 20 μg/mL AuNR-Glu irradiated by a 1064 nm laser with different power densities for 5 min. Mean ± standard deviation, N = 3, * *p* < 0.05, ** *p* < 0.01, *** *p* < 0.001, and **** *p* < 0.0001. (**D**) Fluorescence images after photothermal treatment with 20 μg/mL AuNR-Glu irradiated by a 1064 nm laser with a (**i**,**vi**) 0, (**ii**,**vii**) 0.25, (**iii**,**viii**) 0.5, (**iv**,**ix**) 0.75, and (**v**,**x**) 1.0 W/cm^2^ power density for 5 min. Cells that stably expressed GFP on their tubulin (live: green) were stained with propidium iodide (dead: red) after 24 h post incubation (scale bars: 20 μm). The cells without AuNR-Glu were used as the control [60].

**Figure 4:**
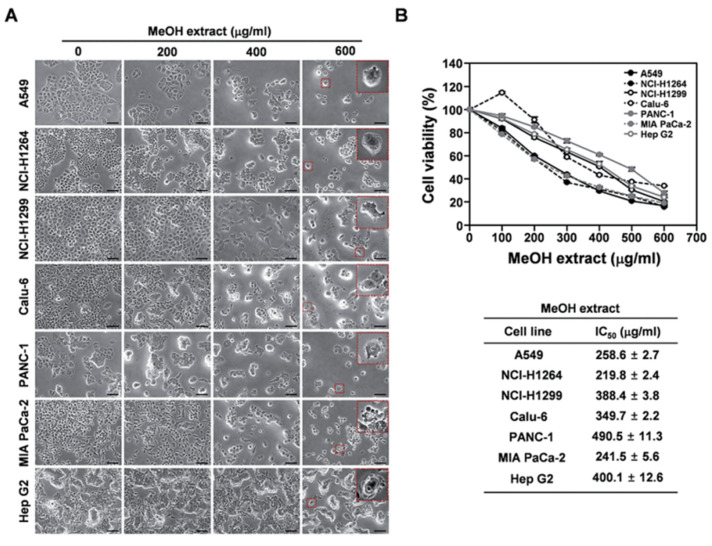
(**A**) Representative bright-field images (200× magnification) of human cancer cells taken at 48 h after treatment with the *P. ravenelii* MeOH extract at the indicated concentrations. Magnified regions are shown as insets. (**B**) Viability of human cancer cell lines after treatment with the *P. ravenelii* MeOH extract at the indicated concentrations (upper panels) and IC_50_ values of the extract for the corresponding cell line (lower panels). Data are representative of three independent experiments (*n* = 3) and are presented as means ± SD. Scale bar: 100 µm [61].

The complex polysaccharides from mushrooms and other resources have been considered a better and effective option for antitumor activity due to their better anticancerous potential. Mushroom-derived polysaccharides can suppress tumor growth by inducing the apoptosis of tumor cells and enhancing immunotherapy and chemotherapy [55,56,57,58]. In another study, Zheng and coworkers isolated the triple-helical β-glucan (THG) from *Lentinus edodes* (shiitake) and examined their antitumor activity in the presence of mouse hepatoma carcinoma cell lines (H22) [59]. They fractionated the isolated THG into nine different weight-average molecular weights (M_w_) by ultrasonication and established a relationship between these different fractions of THG and antitumor activity. A drastic decrease in cell viability of H22 was observed in THG-treated media compared to the control, indicating their antitumor potential. The antitumor potential of the extracted THG is profoundly affected by its molecular weight, and the low-molecular-weight fraction exhibited better antitumor activity than others. The antitumor potential of the extracted THG is also affected by its chain conformations. The low-molecular-weight fraction of THG with high chain stiffness facilitated the effective binding of β-glucan with immune cell receptors and initiated the immune responses. The structural stability and biocompatibility favor the β-glucan to use as a carrier for different nanoparticles in biomedical applications. Li et al. synthesized β-glucan-modified gold nanorods (AuNRs) and evaluated their cytotoxicity in the presence of MCF-7 cell lines [60]. *Β-glucan* was extracted from *Pleurotus tuber-regium* (king tuber) mushroom to modified AuNRs. β-glucan-coated AuNRs (AuNRs-Glu) were prepared by mixing the freshly prepared AuNRs in the aqueous glucan solution with continuous stirring at 85 °C for 30 min. A significant decrease in cell viability of MCF-7 was observed in the presence of AuNRs-Glu at 20 µg/mL and a laser power density of 0.75 W/cm^2^. The developed AuNRs-Glu showed greater colloidal stability in different biological media and simulated gastric fluid. The developed AuNRs-Glu demonstrated low cytotoxicity and more excellent photothermal stability, indicating an effective and suitable photothermal agent for cancer treatment. The enhancement in biocompatibility and photothermal activity was due to the wrapping of excellent biocompatible β-glucan with gold nanoparticles that significantly enhanced the colloidal and photothermal stability of the pure gold nanoparticles. The cell viability of MCF-7 cells in the presence of the synthesized AuNRs-Glu at different concentrations and power density is shown in Figure 3. *Pulveroboletus ravenelii* (Ravenel’s bolete) is an edible mushroom found in China, Japan, Korea, Singapore, and North America. It is commonly used in Traditional Chinese Medicine (TCM) and dye applications. Kim et al. extracted the vulpine acid (VA) from *Pulveroboletus ravenelii* and evaluated their anticancerous potential against various human cancer cells [61]. A substantial decrease in cell viability was observed in the presence of extracted VA, indicating its cytotoxicity against cancer cells. The cytotoxic behavior of the extracted VA against lung, pancreatic ductal, and hepatocellular carcinoma cells is shown in Figure 4. A decrease in cell viability of U937 human monocytic leukemia cells was also observed in *Hericium erinaceus* mushroom extract through the suppression of cell proliferation pathways [62]. The cellular responses of other mushrooms are also summarized in Table 2.

### 4.2. Antioxidant and Antibacterial Activity

**Figure 5:**
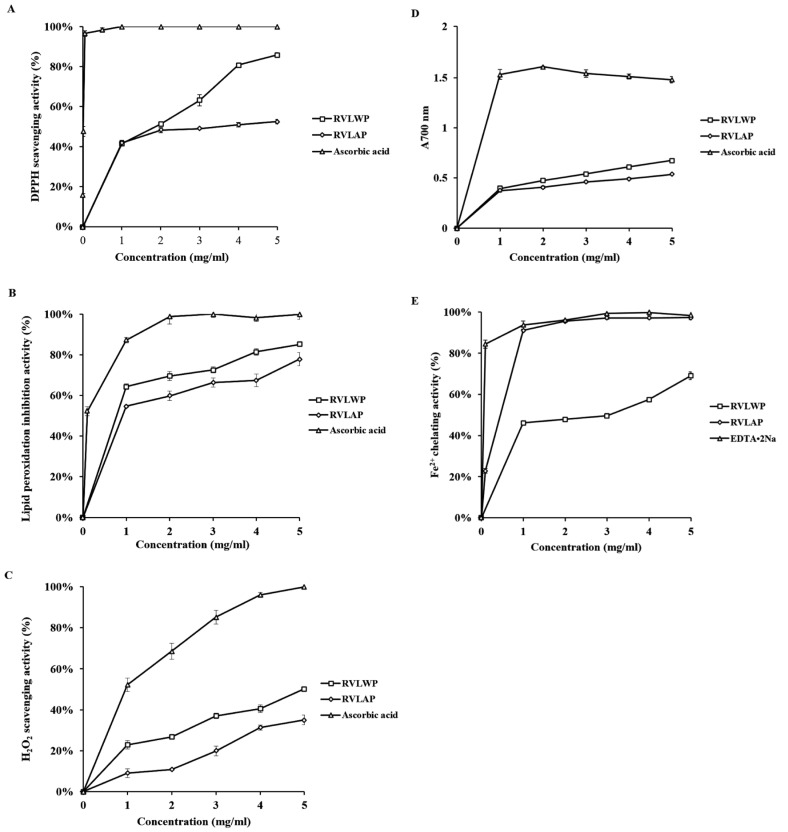
Antioxidant activities of water-soluble polysaccharides (RVLWPs) and alkaline-soluble polysaccharides (RVLAPs): (**A**) scavenging of DPPH radicals; (**B**) inhibitory effect on lipid peroxidation; (**C**) hydrogen peroxide scavenging activity; (**D**) reducing power; (**E**) Fe^2+^ ion-chelating activity. The values are representative of three separate experiments [83]. The results are presented as the mean ± standard deviation (SD) from three independent experiments (*n* = 3).

**Figure 6:**
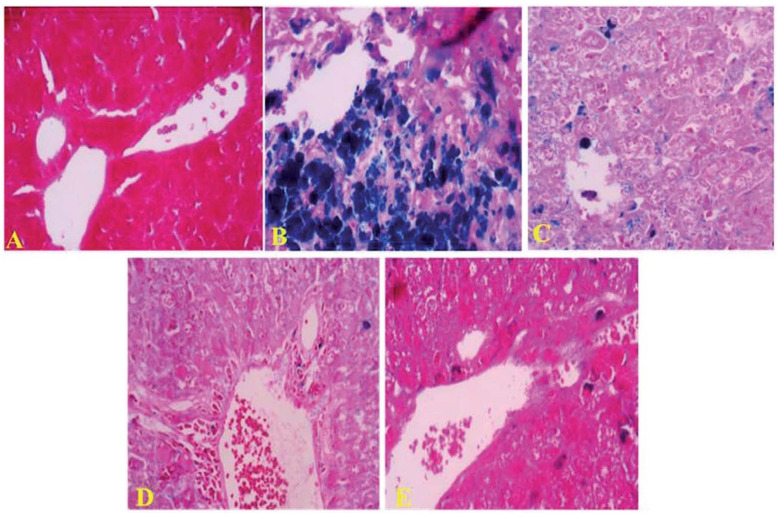
Effects of *Cantharellus cibarius* on iron deposition in mice liver. Representative microscopic photographs of livers stained with Prussian blue (400× magnification). (**A**) Control group, (**B**) iron overloaded group, (**C**) DFO group, (**D**) ethyl acetate extract group, and (**E**) methanolic extract group) [87].

Reactive oxygen species (ROS) have received a great amount of interest owing to their roles in several diseases such as cancer, heart disease, and diabetes [77,78,79,80,81]. Antioxidants protect the cells by the reduction of the reaction in cellular environments. Mushroom-derived polysaccharides exhibited free radical scavenging activity and can be applied as antioxidant materials for human health [82]. Liu and coworkers isolated water-soluble polysaccharides (RVLWPs) and alkaline-soluble polysaccharides (RVLAPs) from *Russula vinosa* and assessed their antioxidant and hepatoprotective activities on carbon tetrachloride (CCl_4_)-activated acute damage liver in mice [83]. RVLWP and RVLAP extracts showed remarkably better scavenging activity. Their radical scavenging activity (IC_50_) values in 1,1-diphenyl-2-picrylhydrazyl (DPPH) were 1.55 ± 0.004 and 3.37 ± 0.21 mg/mL for RVLWP and RVLAP, respectively. The radical scavenging activity (IC_50_) values in hydrogen peroxide were 6.07 ± 0.24 and 9.23 ± 0.54 mg/mL for RVLWP and RVLAP, respectively. Antioxidant activities of RVLWP and RVLAP extract are represented in Figure 5. Petrovic et al. extracted the chemical constituents from an edible *Laetiporus sulphureus* mushroom and studied their proximate composition, antioxidant efficiency, and antimicrobial potential [84]. The *Laetiporus sulphureus* derived extract exhibited better antimicrobial activity in some cases compared to the used antibiotics and mycotic. Therefore, this edible mushroom can be used as natural antioxidants or antimicrobial agents with high nutritional value and health benefits [85]. Tuberculosis (TB) is one of the severe diseases caused by *Mycobacterium tuberculosis*, affecting 2–3 million people per year. Isoniazid and rifampicin drugs are commonly used in the treatment of TB. However, isoniazid and rifampicin drugs demonstrated hepatotoxic activity. Therefore, it is necessary to develop more effective and alternative options from natural resources. It is well-known that Basidiomycetes mushrooms contain several bioactive compounds, including anti-TB agents. Arpha et al. isolated the chemical constituents from an edible *Astraeus odoratus* mushroom and evaluated their antibacterial activity, as well as cytotoxicity against H_37_Ra (*Mycobacterium tuberculosis*) and cancer cell lines, respectively [86]. *Astraeus odoratus* extract showed improved antibacterial and cytotoxic activity, indicating their potential to be used as an alternative supplement for antibacterial and anticancerous applications. The ion-chelation potential of the materials provides an alternative and attractive approach to remove the excess ions from body fluids. Khalili and coworkers extracted the chemical constituents from an edible *Cantharellus cibarius* mushroom and evaluated their iron-chelation activity in mice [87]. The extracted material exhibited the superior potential of free iron removal from the liver of mice due to their better chelation efficiency. The effects of *Cantharellus cibarius* extract for removing the deposited iron in the liver of mice are shown in Figure 6.

### 4.3. Immunomodulatory Activity

Immunomodulators play an important role in the immunity enhancement of the individual against different diseases. The individual immune system produces a variety of immunomodulatory to maintain the homeostasis of the living organism. They are categorized into immunosuppressants, immunostimulants, and immunoadjuvants [88,89,90,91]. Mushroom-derived bioactive components can stimulate both innate and adaptive immune system activities of the individual. The bioactive components proliferate and stimulate the innate immune system, such as neutrophils, natural killer (NK) cells, macrophages, and cytokine expression. The secreted cytokines trigger the adaptive immune system via the elevation of B cells for antibody generation, followed by the stimulation of T-cell differentiation to T helper (Th-1) and Th-2 cells, leading to the enhancement of the immunities. The high molecular weight of polysaccharides restricts them to direct penetration to the immune cells. Therefore, the trigger process of polysaccharides involves different cell receptors, and their efficiency is profoundly affected by their binding affinity to the receptors [88,89,90,91,92]. The immunomodulatory activities of the commonly used mushroom extract are summarized in Table 3. The different active constituents of the mushroom, such as β-glucan, protein complexes, and others, can stimulate the cellular functions to secrete the different kinds of factors for the macrophage activation, leading to the enhancement in the immunomodulatory or antitumor activities of the mushroom. Khursheed et al. also summarized the therapeutic potentials of different mushrooms [93].

## 5. Safety Assessment of Mushrooms

Mushrooms have gained significant popularity to be used as a dietary supplement due to different bioactive components. However, it has been seen that several medicinal mushrooms are not suitable for human health, and toxic substances occur in some medicinal mushrooms [116,117]. Therefore, it is necessary to examine the toxicological effects of mushrooms before using them as a dietary supplement. As per World Health Organization (WHO) reports, approximately 0.346 million people have died since 2004 globally, and the primary reason is mushroom poisoning [118]. Various mushroom poisoning cases are reported in different countries per year. Mushroom toxicity is categorized into seven main groups: amatoxins, orellanus, gyromitrin, muscarine, ibotenic acid, psilocybin, and coprine. Amatoxins are bicyclic octapeptides and strong toxic substances. Amatoxins inhibited the DNA-dependent RNA polymerase activity, leading to a reduction in protein synthesis, and cell necrosis or apoptosis [119]. Mushrooms having coprine cause the addiction and accumulate in the blood when consumed with alcohol. A summary of different toxic substances present in mushrooms with their potential toxicity is given in Table 4.

## 6. Conclusions

Mushrooms have received significant attention from the scientific community due to their high nutritional and medicinal values. They are used as a food supplement and induce the immunity of individuals through their different bioactive components. A decrease in the number of *Mycobacterium tuberculosis* bacteria in the presence of mushroom extract provides a new approach in the treatment of TB disease, instead of the commercially available medicines, which have cytotoxicity. A significant decrease in the cell viability of tumor cells was observed in the presence of mushroom extract, indicating their antitumor activity. Therefore, mushroom extracts have the potential to use as a food supplement for therapeutic applications.

## Figures and Tables

**Table 1:** A scientific and common name of mushrooms and their significant constituents [15]. (GLPS = Ganoderma lucidum polysaccharide; PSP = polysaccharide peptide; PSK = polysaccharide K.).

Scientific Name	Common Name	Specific Constituent	Type of Constituent
*Agaricus blazei*	*Agaricus*	β-d-glucan	Polysaccharide
*Ganoderma lucidum*	Reishi, lingzhi	Ganoderic acid	Protein
		Danoderiol	Protein
		Danderenic acid	Protein
		Lucidenic acid	Protein
		GLPS	Polysaccharide
*Cordyceps sinesis*	*Cordyceps*, caterpillar mushroom	Adenosine	Nucleotide
		Cordycepin	Nucleotide
*Trametes versicolor* (formerly *Coriolus versicolor*)	Turkey tail	PS	Polysaccharide peptide
		PSK	Polysaccharide peptide
*Grifolia frondosa*	Maitake	Grifolan	Polysaccharide
		D-fraction	
		MD-fraction	Polysaccharide

**Table 2:** The effects of different mushrooms on the cellular response.

Mushroom	Model	Compound	Response	Reference
Button	In vivo	Hot water extract	Upregulation of CD69, CD49 T, CD3, CD19, NK activity, phagocytosis of spleen cells, and decrease the liver and spleen weight	[63,64]
			Enhancement in the level of the interleukin (IL) IL-1, IL-6, TNF-α, and IL-1β	[26,27]
Maitake	In vivo	Extract	Increased cell growth and differentiation	[65]
			Upregulation of tumor-specific CD8+, CD4+ T cells, NK activity, T-cell infiltration, and decrease the Treg cells activity	[50]
Reishi	In vivo	Polysaccharides and extract	Enhancement of CD56, NK cells, phagocytosis, CD8+, CD4+ cells activity, and decrease the cancerous cell activities such as cell growth, and adhesion	[66,67,68]
Cordyceps	In vitro	Cordycepin	Decreases the activity of matrix metallopeptidase 9 (MMP-9), and NF-κB protein complex	[69]
			Inhibition of TNF-α, IL-1β, NO, prostaglandin (E_2_)	[30]
Turkey Tail	In vivo	Powder	Increase lymphocyte count, NK activity, CD8+ T cells, and CD19+ B cells activity	[70]
Shiitake	In vitro	Extract	Inhibition of chicken tumor cells growth and increased the secretion level of interferon-α (IFN-α), IL-1β, IL-6, IL-12, IL-15, IL-18, and tumor necrosis factor superfamily 15 (TNFSF15)	[71]
Cracked-cap Polypore	In vitro and vivo	Extract	Suppressed the growth of tumor cells	[72]
Black Hoof			Enhancement in cell proliferation, migration, tube formation and phosphorylation of vascular endothelial growth factor receptor (VEGFR)-2	[73]
*Pleurotus florida*	In vitro	Extract	Anti-inflammatory and antiplatelet-aggregating potential	[74]
*Albatrellus confluences*	In vitro	Extract	Antitumor activity and upregulation of death-associated protein kinase 1 (DAPK1)	[75,76]

**Table 3:** Mushrooms extract exhibiting immunomodulatory and other activities.

Mushroom Species	Active Materials	Functions	Reference
*Agaricus bisporus*	Monounsaturated fatty acids and ergosterol	Antioxidant and antimicrobial properties	[93,94]
*Agaricus brasiliensis*	Polyunsaturated fatty acids		
*Agaricus bisporus*	Lectins	Immunomodulatory	
*Agaricus blazei*	Polysaccharides		
*Phellinus linteus*	Polysaccharides	Immunomodulatory	[95,96,97]
*Phellinus igniarius*			
*Phellinus sp.*		Antiaging activity	
*Ficus carica*	Polysaccharides	Immunomodulatory activity	[98]
*Pleurotus eryngii*	Protein/polyphenols	Immunomodulatory and anti-inflammatory	[99,100,101,102,103,104,105]
*Pleurotus tuber-regium*	Polysaccharides	Immunomodulatory and antitumor	
*Pleurotus citrinopileatus*	Protein PCP-3A	Immunomodulatory and anti-inflammatory	
*Pulveroboletus ravenelii*	Vulpinic acid	Anticancerous	[105]
*Prunella vulgaris*	Heteropolysaccharides	Immunomodulatory	[106]
*Lentinula edodes*	Polysaccharides	Immunomodulatory	[107]
*Talinum triangulare*	Polysaccharides	Antitumor efficacy	[108]
*Tremella fuciformis*	Berk extract	Anti-inflammatory	[109]
*Antrodia camphorata*	Protein	Immunomodulatory	[110]
*Poria cocos*	Protein	Immunomodulatory	[111]
*Ganoderma atrum*	Protein	Anticancerous	[112]
*Tricholoma matsutake*	α-Glucan–protein complex	Immunomodulatory	[113]
*Hericium erinaceus*	Polysaccharides	Immunomodulatory	[114]
*Flammulina velutipes*	Protein	Anticancerous	[115]

**Table 4:** Clinical manifestations, action sites, and toxicity potential of the indicated mushrooms (GIT, gastrointestinal tract; ANS, autonomic nervous system; CNS, central nervous system).

Species	Toxic Substance	Action Sites	Toxic Syndrome	Mortality Rate	Reference
*Amanita phalloides* *Amanita verna* *Amanita bisporigera* *Amanita virosa*	Amatoxins, phallotoxins	Live, kidney, GIT	Delayed liver toxicity and gastroenteritis	~2–30%	[120]
*Cortinarius orellanus* *Cortinarius speciosissinus*	Orellanine	Kidney, GIT	Delayed renal failure	Very less	[121]
*Gyromitra esculenta* *Gyromitra infula* *Chrysina macropus*	Gyromitrin	Liver, blood, GIT, CNS	Delayed gastroenteritis and liver toxicity	0–10%	[122]
*Clitocybe dealbata* *Clitocybe illudens* *Boletus calopus*	Muscarine	ANS	Vomiting, salivation, diarrhea	Very less	[123]
*Amanita muscaria* *Amanita pantherina* *Amanita gemmata*	Ibotenic acid	CNS	Hallucinations, seizures, agitation	Very less	[124]
*Psilocybe cubensis* *Psilocybe Mexicana* *Conocybe cyanopus*	Psilocybin	CNS	Hallucinations	Very less	[125]
*Coprinus atramentarius*	Coprine	-	Anxiety, headache, chest pain	Very less	[126]

## Data Availability

Not applicable.
